# A prospective study in male recipients of kidney transplantation reveals divergent patterns for inhibin B and testosterone secretions

**DOI:** 10.1186/2051-4190-24-11

**Published:** 2014-06-16

**Authors:** Safouane M Hamdi, Marie Walschaerts, Louis Bujan, Lionel Rostaing, Nassim Kamar

**Affiliations:** Groupe de Recherche en Fertilité Humaine, EA 3694, Hôpital Paule de Viguier, Toulouse, F-31000 France; Université Paul-Sabatier, Toulouse, F-31000 France; Département de Néphrologie et Transplantation d’Organes, CHU Toulouse, Toulouse, F-31000 France

**Keywords:** Dialysis, Kidney transplantation, Gonadotropins, Testosterone, Inhibin B, Immunosuppression, Dialyse, Transplantation rénale, Gonadotrophines, Testostérone, Inhibine B, Immunusuppresseurs

## Abstract

**Background:**

Male patients with chronic kidney disease often exhibit the biological and clinical hallmarks of an abnormal hypothalamo–pituitary–gonadal axis. It is known that dialysis does not reverse this impaired endocrine status; however, the impact of kidney transplantation (KT) is still controversial. The aim of our study was to investigate the levels of serum gonadotropins, testosterone, and inhibin B during dialysis and after KT.

**Methods:**

A longitudinal and prospective single center study was led in an academic setting. Blood hormones levels were assayed by immunoassays in 53 men (mean age: 37 years) receiving dialysis (T_0_) and at 6 months post-KT (T_180_). These data were compared with those from 46 fertile semen donors (mean age: 37 years). The main outcome measure was the between-groups differences in hormones levels. A second criterion was the comparison of T_0_ and T_180_ hormones levels according to the immunosuppressive regimen.

**Results:**

For patients ongoing dialysis, luteinizing hormone (LH) and follicle-stimulating hormone (FSH) mean levels were high, whereas testosterone and inhibin B mean levels remained normal. After KT, LH levels returned to normal whereas FSH was significantly increased. Testosterone levels remained normal whereas inhibin B levels significantly decreased. We found that the combination tacrolimus plus mycophenolic acid significantly decreased post-KT inhibin B levels. Moreover, we found that pre-graft inhibin-B level was independent of testosterone and could predict low post-operative inhibin B level with a sensitivity of 77% and a specificity of 92%.

**Conclusions:**

Our study suggests that endocrine secretions of Leydig and Sertoli cells are differently impacted by dialysis, KT and immunosuppressive regimen raising new issues to explore.

## Abstract (fr)

**Contexte:**

Les hommes insuffisants rénaux chroniques présentent souvent des signes cliniques et biologiques d’une atteinte de l’axe hypothalamo-hypophyso-testiculaire. Il est bien établi que la dialyse n’améliore pas ces anomalies endocriniennes mais l’impact de la transplantation rénale reste encore controversé. Le but de l’étude est d’explorer les taux circulants des gonadotrophines, de la testostérone et de l’inhibine B durant la période de dialyse et après la greffe de rein.

**Patients et Méthodes:**

Cette étude longitudinale et prospective est réalisée dans un centre hospitalo-universitaire. Les taux sériques des hormones sont mesurés par immunodosage chez 53 patients (âge moyen : 37 ans) durant la période de dialyse (T0) et 6 mois après la greffe rénale (T180). Ces données sont comparées à celles de 46 donneurs de sperme fertiles (âge moyen : 37 ans). Le principal critère de jugement est la différence des taux hormonaux entre les trois groupes. Le critère secondaire est la comparaison des taux pré- et post-greffe en fonction du traitement immunosuppresseur.

**Resultats:**

Durant la période de dialyse, les taux moyens de LH et de FSH sont élevés alors que ceux de testostérone et inhibine B sont normaux. Après la greffe, la LH revient à la normale alors que la FSH augmente. Les taux de testostérone restent normaux mais ceux d’inhibine B augmentent significativement. Par ailleurs, l’étude montre que la combinaison tacrolimus + mycophénolate diminue clairement les taux post-greffe d’inhibine B. Elle montre également que les taux pré-greffe d’inhibine B sont indépendants de ceux de la testostérone et qu’ils peuvent prédire les taux bas d’inhibine B avec une sensibilité de 77% et une spécificité de 92%.

**Conclusions:**

L’étude suggère que les sécrétions endocrines des cellules de Leydig et de Sertoli sont impactées de manière différente par la dialyse, la transplantation rénale et le traitement immunosuppresseurs ouvrant ainsi de nouvelles perspectives d’investigation.

## Background

Abnormalities of the male hypothalamo–pituitary–gonadal (HPG) axis during end-stage renal disease are well recognized, including those of testicular function: i.e., steroidogenesis and spermatogenesis [1–3]. Unfortunately, these abnormalities are not reversed in uremic patients under regular dialysis and can induce hypogonadism, altered spermatogenesis, low libido, erectile dysfunction, and infertility [4–12]. Moreover, hypogonadism could be linked to an increased risk of premature cardiovascular disease and mortality [13, 14].

It is now realized that, after a successful KT, hypothalamo–pituitary and testicular dysfunctions may recover, but the extent of this recovery and the proportion of grafted patients that do recover is still controversial [3, 10]. It is still not possible to predict the future testicular function of a uremic patient after a KT.

Inhibin B is a glycoprotein hormone of gonadal origin, which consists of two dissimilar subunits (α and β_B_) linked by a disulfide bond. It is produced by Sertoli cells in the seminiferous tubules and is the only form of inhibin present in men. Follicle-stimulating hormone (FSH) is a potent stimulator of inhibin B production, which, in turn, regulates FSH in a negative feed-back mechanism. It is well-established that serum levels of inhibin B are positively correlated with sperm concentration, total sperm count, and testicular volume. High serum levels of inhibin B are observed in normal and fertile individuals whereas significantly lower serum levels of inhibin B are reported in men with testicular dysfunction [15]. Thus, inhibin B has been considered a direct marker for Sertoli-cell function and an indirect marker for spermatogenesis. For the above reasons, inhibin B has been used during the last two decades to categorize male infertility in epidemiological studies [16, 17]. Recently, inhibin B has been proposed as a valuable biomarker for human testicular toxicity [18, 19]. In this setting, it would be of great interest to investigate the serum levels of inhibin B in men receiving dialysis and/or after a KT, as this issue has been scarcely addressed in the literature. Our first objective was to investigate the levels of circulating pre- and post-KT gonadotropins, testosterone, and inhibin B in men aged <50 years, and to compare these data with those from fertile semen donors. Second, we analyzed the effect of immunosuppressive regimen on testosterone and inhibin B secretions. Finally, we researched which factors could predict post-operative inhibin B levels in order to foresee the impact of a KT on spermatogenesis and the patient’s fertility.

## Methods

### The patients

This longitudinal and prospective study was performed at the organ-transplant unit of Toulouse University Hospital between May 2006 and March 2008. This study was approved by the ethics committee of the Universitary Hospital of Toulouse and written consents were obtained from all patients or their guardians, as well as from semen donors. During this period, 257 patients received a KT. Eligibility criteria are underlined below: of these graft receivers, 155 were male and, amongst them, 87 were aged >18 and <50 years. We selected 53 patients whose blood-hormone assays were available on the day of transplantation (T_0_) and at 6 months after (T_180_). Baseline clinical data, including age, duration of dialysis, and body-mass index (BMI), were collected from each patient. At discharge, the immunosuppressive regimen included corticosteroids and were as follows: 19 patients received tacrolimus (TAC) plus mycophenolic acid (MPA), 18 received cyclosporine A (CsA) plus MPA, nine received TAC plus the mammalian target of rapamycin (mTORi), four received belatacept plus MPA, and three patients received mTORi plus MPA. No specific or additional treatments were administered to patients for the purpose of the study and data were collected by reviewing medical charts.

### The control population

The control population consisted of 46 semen donors recruited from the department of male infertility at Toulouse University Hospital between December 2006 and May 2011. All donors who gave their informed consent and had provided a blood sample were included in the study. The protocols for selecting semen donors have been reported elsewhere [20]. Briefly, all donors had previously fathered at least one child and those aged <20 or >45 years, or who had an infertile brother, were excluded. No specific or additional treatments were administered to donors for the purpose of the study and data were collected by reviewing medical charts.

### Assessment of blood hormones

Venous blood samples were obtained in the morning as following: for patients, samples were collected just before the transplantation (T_0_) and during the routine visit of the 6^th^ month post-KT (T_180_); for semen donors the blood sample was collected during the routine clinical examination. Analyses were performed in the clinical biochemistry department of the hospital. Serum hormone levels (LH, FSH, total testosterone) were assessed by an automated immunoassay (Centaur XP of SIEMENS). For a general population of adult males, the laboratory reference ranges were 1.5–9.3 IU/L for LH; 1.4–11 IU/L for FSH, and 288–820 ng/dL for total testosterone. The inter- and intra-coefficients of variation (CVs) were <3% for both LH and FSH and, respectively, 4% and 7% for total testosterone. Serum inhibin B levels were quantified in duplicate using a manual ELISA protocol from Oxford Bio Innovation, with a normal range for adult males of between 80 and 320 pg/mL [21]. The intra- and inter-CVs were, respectively, 3.5% and 10.2%. The sensitivity of the assay was set at 15 pg/mL, which was the concentration that corresponded to the lowest point on the standard curve. Hence, for the statistics, all serum samples with an inhibin B concentration of <15 pg/mL were given an arbitrary value of 14 pg/mL. Since the study was led in a reproductive endocrinology setting that considers blood hormones as indirect biomarkers of semen parameters, their cutoffs were carefully selected. For LH and FSH, the cutoffs of hypergonadotrophic status were set, respectively, at 8 IU/L [22] and 7 IU/L [23]. Testosterone deficiency was defined as a total serum testosterone level <288 ng/dL (<10 nmol/L), in accordance with the recent international guideline from the Endocrine Society [24] and the cut-off point for diminished Sertoli-cell secretion of inhibin B and indirect marker of impaired spermatogenesis was set at 80 pg/mL [25, 26].

### Statistical analyses

Comparisons between the groups of patients were performed according to a normality pattern, using the Kolmogonov–Smirnov test. If the normality test was positive, the Student’s t-test and a paired t-test were used for unpaired and paired groups. If the normality test was negative, the Mann–Whitney and Wilcoxon rank tests were used. Comparison of several groups was done using ANOVA, the Holm–Sidak test, and an ANOVA test of ranks. We used the classification and regression-tree method [27] to predict the post-KT inhibin-B levels of our overall data. The number of missing data is reported in Table 1. Cases and controls were not matched, an overall comparison was performed. Statistical significance was set at *p* <0.05. Statistical analyses were performed using SigmaStat 3.5 and R packages.

**Table 1:** **Clinical, biological and hormonal status of donors and patients**

	A–healthy controls (n = 46)	B–graft recipients before transplantation (n = 53)	C–graft recipients after transplantation (n = 53)	p value		
				B vs A	B vs C	C vs A
**Age** (*years*)^a^	36.8 (*5.3*)	37.0 (*8.0*)	–	0.73^d^		
**BMI** ^a^	–	22.6 (*4.0*)	23.4 (4.1)		0.33	
**HD duration** (*months*)^a^	–	35.7 (*28.6*)	–			
**Creatinine** (*μmol/L*)^a^	–	846.0 (*221.0*)	141.0 (*79.0*)		<0.001	
**GFR** (mL/min)^b^, **LH** (*IU/L*)			57.1 (17.2)			
mean, (SD)	3.3 (*1.3*)	8.9 (*6.5*)	6.7 (*5.0*)	<0.001	<0.001	<0.001
median, (5^th^ – 95^th^ percentile)	3.1 (*1.5–5.8*)	6.3 (*3.1–24.8*)	5.1 (*2.0–16.4*)			
value > 8 UI/L^c^, (%)	0 (*0.0*)	19 (*36.5*)	13 (*24.5*)			
missing data^c^, (%)	1 (*2.2*)	1 (1.9)	0 (*0.0*)			
**FSH** (*IU/L*)						
mean, (SD)	3.7 (*1.7*)	7.7 (*7.6*)	11.3 (*8.2*)	0.001	<0.001	<0.001
median, (5^th^–95^th^ percentile)	3.3 (*1.4–7.0*)	4.9 (*1.6–27.5*)	9.7 (*2.0–29.1*)			
value > 7 UI/L^c^, (%)	1 (*2.2*)	19 (*35.8*)	34 (*65.4*)			
missing data^c^, (%)	1 (*2.2*)	0 (*0.0*)	1 (*1.9*)			
**Testosterone** (*ng/dL*)						
mean (SD)	512 (*156*)	447 (*213*)	398 (*151*)	0.12	0.12	<0.001
median (5^th^–95^th^ percentile)	522 (*261*–*800*)	432 (*171*–*811*)	371 (*190*–*618*)			
value < 288 ng/dL^c^, (%)	2 (*5.2*)	12 (*22.6*)	13 (*24.5*)			
missing data^c^ (%)	8 (*17*)	5 (*9.4*)	3 (*5.2*)			
**Inhibin B** (*pg/mL*)						
mean (SD)	149 (*71*)	145 (102)	93.6 (79.8)	0.48	<0.001	<0.001
median (5^th^–95^th^ percentile)	137 (*56*–*309*)	127 (14–348)	68 (14–280)			
value < 80 pg/mL^c^, (%)	7 (*15.2*)	12 (27.9)	25 (47.2)			
missing data^c^, (%)	0 (*0.0*)	10 (18.9)	8 (15.1)			

Data were collected from 46 healthy semen donors and 53 male recipients before (T_**0**_) and at 6 months (T_**180**_) after a kidney transplant.

^a^Mean (SD), ^b^glomerular filtration rate using the MDRD formula [28], ^c^number of events, ^d^statistical significance is set at p < 0.05.

## Results

### Assessment of blood hormones

In a first approach, we performed overall analyses of the patients’ and semen donors’ characteristics. Table 1 summarizes their main clinical and biological data as well as their hormonal status and we report herein some meaningful results (as mean+/− SD). Semen donors and graft recipients did not differ in age (36.8 ± 5.3 vs. 37 ± 8 years, respectively, p = 0.73). Prior to a KT, the patients underwent an average of 35.7 ± 28.6 months of dialysis. At 6 months after transplantation, mean serum creatinine levels and glomerular-filtration rate (GFR) were 141 ± 79 μmol/L and 57.1 ± 17.2 mL/min, respectively.

#### Gonadotropins

Before a KT, mean LH level of the KT patients (8.9 IU/L) slightly exceeded the cutoff and was significantly higher than the semen donors (3.3 IU/L, p < 0.001). Nineteen patients (36.5%) had a LH level above the normal range. Postoperatively, that level decreased significantly (6.7 IU/L, p < 0.001), but still remained higher than that of the male controls (3.3 IU/L, p < 0.001). During dialysis, mean FSH level of the patients (7.7 IU/L) was slightly above the cutoff and was significantly greater than that of the semen donors (3.7 IU/L, p = 0.001). Nineteen patients (35.8%) exhibited abnormally high FSH levels. At 6 months after a KT, FSH levels had increased significantly (11.3 IU/L, p < 0.001), with 34 patients (65.4%) exhibiting levels above the cutoff.

#### Testosterone

Prior to a KT, mean testosterone level of the KT patients (447 ng/dL) was not significantly different from that of semen donors (512 ng/dL, p = 0.12). However, the former were more often hypogonadal (22.6% vs*.* 5.2%). After transplantation, the mean testosterone level (398 ng/dL, p = 0.12) as well as the number of hypogonadal patients (24.5%) had not significantly changed. Strikingly, the postoperative mean testosterone level of patients remained significantly lower than that of donors (p < 0.001).

#### Inhibin B

Just before a KT, the mean inhibin B level of patients (145 pg/mL) did not vary from that of semen donors (149 pg/mL, p = 0.48). By 6 months after a KT, inhibin B level had significantly decreased (p < 0.001): almost half of the patients had a serum level <80 pg/mL. Moreover, the mean inhibin-B level of these graft recipients (93.6 pg/mL) remained siginificantly below that of semen donors (p < 0.001).

### Intra-individual variations in testosterone and inhibin-B secretions

**Figure 1:**
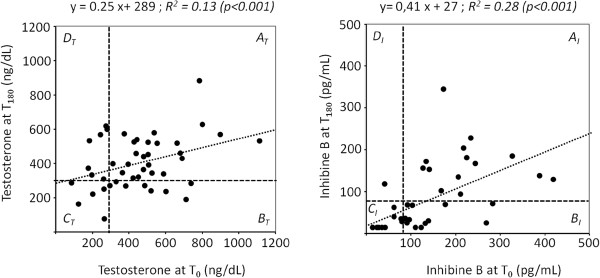
**Linear regression of individual testosterone (panel a, n = 45) and inhibin-B (panel b, n = 36) levels before (T**_**0**_ **) and at 6 months after (T**_**180**_ **) transplantation.** Dashed lines represent the lower limit of the normal ranges: testosterone, 288 ng/dL; inhibin B, 80 pg/mL. Patients were then distributed into four subgroups (secretion before/after graft): A_T_ and A_I_ (normal/normal), B_T_ and B_I_ (normal/low), C_T_ and C_I_ (low/low), and D_T_ and D_I_ (low/normal).

In a second step, and according to the semiologic value of testosterone and inhibin B blood concentrations, we focused on the intra-individual variations before and after a KT. For both hormones, pre- and post-KT levels were significantly correlated (Figure 1). However, in a separate analysis, using linear regression, we found that inhibin B and testosterone levels before and after a KT were not correlated (data not shown): this suggests that both endocrine testicular secretions were independent. By focusing on the lower limit of testosterone and inhibin B blood levels (dashed lines), we divided each scatter plot into four quadrants, each representing a specific subgroup of patients. We identified those whose hormones levels i) remained normal (quadrants A_T_ and A_I_) ; ii) had dropped from normal to low levels (B_T_, B_I_); iii) had remained below the normal ranges (C_T_, C_I_); and had recovered to within the normal range (D_T_, D_I_).

Analysis of individual testosterone levels revealed that 60% of patients with a graft remained eugonadal (A_T_) whereas 9% remained hypogonadal (C_T_). The rest of the patients could be split into two equal groups (15.5% each): those that had a worsening androgenic status and had become hypogonadal (B_T_), and those that had recovered and had become eugonadal (D_T_). Overall, the majority of patients (34 out of 45) were eugonadal after a KT.

We found a different distribution with regards to inhibin B levels: 38.8% of patients retained normal hormone levels (A_I_) whereas 33.3% had levels that were below the normal range (B_I_). For the other patients, 25% retained a low level of inhibin B after transplantation (C_I_) and only one patient recovered a normal concentration (D_I_). Thus, the majority of KT patients (21 out of 36) exhibited low inhibin-B levels.

Overall, the patients’ distribution among the four secretion profiles, A, B, C, and D, were not the same for both hormones. These data suggest that Leydig and Sertoli cells were differently and independently impacted by transplantation. In order to confirm this hypothesis, we focused on the larger subgroup of patients, i.e., those that remained eugonadal after transplantation (A_T_). We found that inhibin B profiles (A_I_, B_I_, and C_I_) were equally distributed among this subgroup, with, respectively, seven, eight, and seven patients. Despite the small sizes of the subgroups, we could compare their clinical and biological data before a KT. There was no difference in age, duration of dialysis, or testosterone levels between the three profiles (data not shown). However, mean inhibin-B levels at T_0_ did significantly differ with respectively (±SD): 241 (±89), 135 (±67) and 42 (±25) pg/mL (p < 0.001). Hence, in the context of preserved testosterone secretion, the inhibin B profiles did not rely on specific features apart from its own initial level. This finding suggests again an independent impact of KT on Leydig and Sertoli cells.

### Impact of the immunosuppressive regimen on post-KT testosterone and inhibin B levels

In a third step, we determined the hormonal status of patients according to their immunosuppressive regimen (Table 2). We focused on the three main subgroups: CsA plus MPA (*n* = 18), TAC plus MPA (*n* = 19), and TAC plus mTORi therapy (*n* = 9). There were no differences in age, dialysis duration, creatinine, or post-KT GFR between the three subgroups. Whatever the treatment, the patients remained eugonadal after a KT, suggesting that these three regimens did not affect Leydig-cell secretion.

**Table 2:** **Clinical, biological, and hormonal data of patients at T**_**0**_ **and T**_**180**_ **according to immunosuppressive regimen**

	CsA + MPA (***n*** = 18)		TAC + MPA (***n*** = 19)		TAC + mTORi (***n*** = 9)	
	T_0_	T_180_	T_0_	T_180_	T_0_	T_180_
**Age** (years)^a^	36.6 (5.3)		37.6 (8.5)		36.6 (7.9)	
**Time on dialysis** (months)	35.1 (27.3)		36.4 (27.4)		42.0 (38.6)	
**Creatinin** (μmol/L)	822 (223)		844 (209)		854 (167)	
**GFR** (mL/min)^b^		56.3 (15.0)		52.5 (19.8)		67.1 (17.8)
**Testosterone** (ng/dL)	493 (205)	392 (92)	415 (212)	380 (181)	465 (272)	445 (129)
*p*-value (T_0_ vs. T_180_)		*0.118* ^c^		*0.605* ^c^		*0.845* ^c^
**Inhibin B** (pg/mL)	172 (72)	150 (158)	106 (85)	46.8 (50.0)	216 (140)	99.4 (57.5)
*p*-value (T_0_ vs. T_180_)		*0.703* ^c^		***0.026*** ^*d*^		*0.093* ^d^

^a^Mean (SD), ^b^using the MDRD formula [28], ^c^paired Student’s t-test, ^d^Mann–Whitney test.

Under the CsA plus MPA therapy, the mean level of inhibin B did not vary between T_0_ and T_180_, remaining within the normal range (respectively, 172 vs 150 pg/mL, *p* = 0.7). For the TAC plus MPA regimen, the mean inhibin B level before KT was low and decreased significantly at post-KT (respectively, 106 and 46.8 pg/mL, *p* = 0.026). Under TAC plus mTORi therapy, the mean inhibin B level diminished from 216 to 99 pg/mL, but this difference was not significant (*p* = 0.09).

### Prediction of post-operative FSH and inhibin B levels

**Figure 2:**
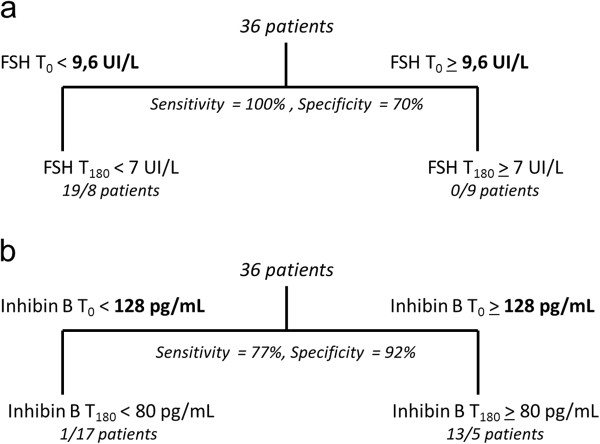
**Regression-tree analysis. a**, FSH. A cutoff of 9.6 IU/L at T_0_ can predict post-transplantation levels with a sensitivity of 100% and a specificity of 70%. **b**, inhibin B. A cutoff of 128 pg/mL at T_0_ can predict post-transplantation levels with a sensitivity of 77% and a specificity of 92%.

Finally, we used a regression-tree algorithm to predict whether post-KT FSH and inhibin-B levels would be below or above their normal levels, respectively 7 IU/L and 80 pg/mL. For this purpose, we focused on patients with a complete set of data (*n* = 36). Regarding FSH, we found that the unique predictive variable was pre-KT FSH level, and that its cutoff value was 9.6 IU/L, with a specificity of 70% and a sensitivity of 100% (Figure 2). For inhibin B, the unique predictive variable was its own pre-KT level with a cutoff value of 128 pg/mL. The sensitivity was lower (77%) than that of FSH but the specificity was higher (92%). This means that a patient with an inhibin B level of <128 pg/mL before transplantation, whatever his age, hormonal status, or immunosuppressive treatment, had a relatively high risk of developing low post-KT levels.

## Discussion

It is now known that, after a successful KT, hypothalamo–pituitary and testicular dysfunctions can recover, but the extent of this recovery and the proportion of grafted patients that do fully recover is still controversial [3, 10]. It is still not possible to predict if the future testicular functions of a uremic patient will be affected after a KT.

In our report, we have explored the hormonal status of 53 patients under dialysis and just prior to a KT. The mean level of testosterone in dialyzed patients was not significantly different from that of the male controls; however, 23% of our patients were within the hypogonadal range, a finding that is consistent with the LH results (Table 1).

Several previous studies have reported that mean testosterone level in dialysis patients was normal or “suboptimal” [5, 7, 9, 11, 12], whereas the percentages of hypogonadal patients varied from 37 to 80%. This discrepancy between studies may result from the small numbers of patients as well as from their ambiguous definition of hypogonadism. For our study, we categorized a man as hypogonadal if his testosterone level was <288 ng/dL (10 nmol/L), which is in accordance with a recent international guideline from the Endocrine Society [24]. The common condition of hypogonadism in dialysis patients, which may be primary or secondary [3], has been linked to several clinical features, such as reduced libido, erectile dysfunction [6, 8, 10, 12, 29], and low ejaculate volume [7, 12].

With regards to inhibin B, we found that median serum levels did not significantly differ between semen donors and dialysis patients. This result seems conflicting with that of FSH levels which were higher in the latter group. However, the standard deviation was higher in dialysis patients, indicating that some subjects exhibited very low levels of inhibin B and the lack of significant difference between the means could be due to the missing data in this group (10/53 for inhibin B levels versus 0/53 for FSH levels, Table 1).

In a further step, we analyzed the impact of the kidney graft on the HPG axis. LH levels decreased significantly whereas those of FSH increased and exceeded the cutoff. Mean testosterone levels did not significantly differ before and after a KT, and the proportion of hypogonadal patients remained stable (25% vs 23%). However, we observed a noteworthy decrease in inhibin B secretion in 47% of graft recipients who had hormone levels below the normal range. This observation is consistent with the increase in FSH after a KT, as reported above. Many studies have described the evolution of the HPG axis before and after a kidney graft [7, 9, 11, 12], and have reported global recovery, but this comparison is difficult to confirm because of the heterogeneity of data (mainly: number and age of patients, dialysis duration, duration of follow-up, hormone assays, and, if detailed, immunosuppressive regimen).

In a last step, we compared patients who had received a graft with control semen donors (Table 1, III vs. I column). Obviously, the four hormone levels were significantly different between the two groups. This indicates that, in the setting of our study, the HPG axis had not fully recovered by 6 months after the KT. The global variation in HPG axis hormones (reported above) hides the fact that a variety of individual patterns can be aggregated into specific categories. This issue has been scarcely addressed in the literature: only two reports have differentiated patients according to their testosterone levels before a KT [10, 11].

In the present report, we first compared pre- and post-KT testosterone levels and identified four secretion profiles (Figure 1). Overall, the patients remained eugonadal after a KT (75%). Moreover, their androgenic status after a KT depended only on preoperative testosterone levels (data not shown). Regarding inhibin B, we identified four profiles (A_I_, B_I_, C_I_, D_I_), which were not distributed between the patients in the same way as those of testosterone. The majority of patients exhibited a low inhibin B level after a KT and only one recovered a normal level. We surmise from these results that both testosterone and inhibin B secretions were independently impacted by the KT. Two additional findings support this hypothesis. There were no correlations between the two hormone levels (data not shown) and, when we explored the individual variations of inhibin B, we found that profiles A_I_, B_I_, C_I_ were equally distributed within the eugonadal group A_T_. Thus, even in the setting of normal testosterone secretion (A_T_), Sertoli cells could react differently at post-KT.

We then compared the effect of immunosuppressive regimens on testis hormones (Table 2). Both calcineurin inhibitors (CsA and TAC), coupled to MPA, did not impair testosterone secretion, a finding that is consistent with previous studies [30, 5]. However, their effect on inhibin-B secretion was different: the mean inhibin B level under CsA plus MPA remained normal whereas it was significantly decreased under TAC plus MPA. Of interest, one study reports that TAC induces morphological alterations of seminiferous tubules and reduces the number of Sertoli cells in rats [31]. However, it is tricky to assess with certainty whether TAC plus MPA are involved in the decreased inhibin-B levels, as the mean preoperative level of this hormone was already low.

It has been previously reported that sirolimus may impair testicular function [32, 33] and male heart transplant recipients treated with sirolimus exhibited a significant increase in FSH levels and a drop of testosterone level [34]. In our study, the TAC plus mTORi regimen did not affect testosterone levels, but reduced those of inhibin B although the decrease was not significant (*p* = 0.093) (with the small number of patients within the subgroup as an acceptable reason). These findings further suggest that the immunosuppressive regimens differently impacted on Leydig and Sertoli cells.

After the study, we wondered whether it was possible to identify predictive variables for post-KT FSH and inhibin B levels. Using regression-tree algorithms, we found that cut-off levels of 9.6 IU/L and 128 pg/mL for FSH and inhibin B, respectively, could predict for abnormal post-KT levels. The specificity of inhibin B cut-off was higher than that of FSH (Figure 2) and may be more helpful to predict testicular impairment. Of interest, this level is still within the normal range (80–320 pg/mL). Because it is well known that dialysis patients have poor semen quality and fertility [35–39] such a normal value is quite puzzling. However, one can assume that uremic patients exhibit, as they do for gonadotropins, reduced metabolic clearance of inhibin B, and that serum levels of this hormone could be artificially high as they are decoupled from spermatogenesis status. Such an interesting hypothesis deserves further investigation.

Despite some positive features, such as the number of included patients, the cross-sectional design, and the use of semen donors as the controls, our study has some limitations. The study is monocentric, the hormonal assessments were made at only 6 months after a KT, and the clinical significance of the variations in testosterone and inhibin B, such as sexual function, spermatogenesis, or fertility, were not assessed. However, this study confirms the controversial status of the HPG recovery after a KT and the need for large, multicentric, prospective, and long-term studies.

## Conclusions

The mains findings of our study are the following: recovery from abnormal testicular function, as observed during hemodialysis, was incomplete at 6 months after a KT; dialysis, KT or immunosuppressive regimen did not impact Leydig and Sertoli cells to the same extent. It also highlights the significance of testosterone and inhibin B assays in this setting and raises new issues into managing the fertility of male transplant patients.

## Abbreviations

CsA: Cyclosporine
FSH: Follicle-stimulating hormone
GFR: Glomerular-filtration rate
HPG: Hypothalamo–pituitary–gonadal
KT: Kidney transplantation
LH: Luteinizing hormone
MPA: Mycophenolic acid
TAC: Tacrolimus
mTORi: Mammalian target of rapamycin inhibitor.
